# A cross-sectional study of factors associated with life satisfaction in Thai elderly

**DOI:** 10.1192/j.eurpsy.2022.271

**Published:** 2022-09-01

**Authors:** S. Joowong, B. Junsirimongkol, P. Khaektao, T. Doungyota, K. Paomee

**Affiliations:** Suansaranrom Psychiatric Hospital, Department Of Mental Health, Suratthani, Thailand

**Keywords:** happiness, Elderly, life satisfaction

## Abstract

**Introduction:**

Aging raises wide-ranging issues within social, economic, welfare, and health care systems. Life satisfaction is regarded as an indicator of the quality of life which, in turn, is associated with mortality and morbidity in older adults.

**Objectives:**

Life Satisfaction is a dimension of happiness and well-being which represents the quality of life in both literacy and every aspect of a person. The purpose of the article is to assess the level of life satisfaction and the factors associated with life satisfaction in old age.

**Methods:**

This research was conducted in a cross-sectional study using 36 items from Satisfaction and Well-being of Elderly (Thai semi-structured in-depth interviews) tools to collect data. The population used in this study was Thai people over 60 and used multistage probability sampling, were held with 2000 elderly individuals from 13 health regions of Thailand.

**Results:**

Of the 2000 samples, the overall life satisfaction was moderate (54.1%). Upon data analysis, ten categories were extracted. However, there are 7 factors that significantly influence the level of life satisfaction of the Thai elderly at p < 0.05: Age, Occupation, Recreational activities, Revenue, Education level, Religious activities, and Social Support. Moreover, when tested with Pearson Correlation found that the relationship between and Thai brief screening for depression (2Q) was low correlated (r -0.121, P=0.000).

**Figure:**
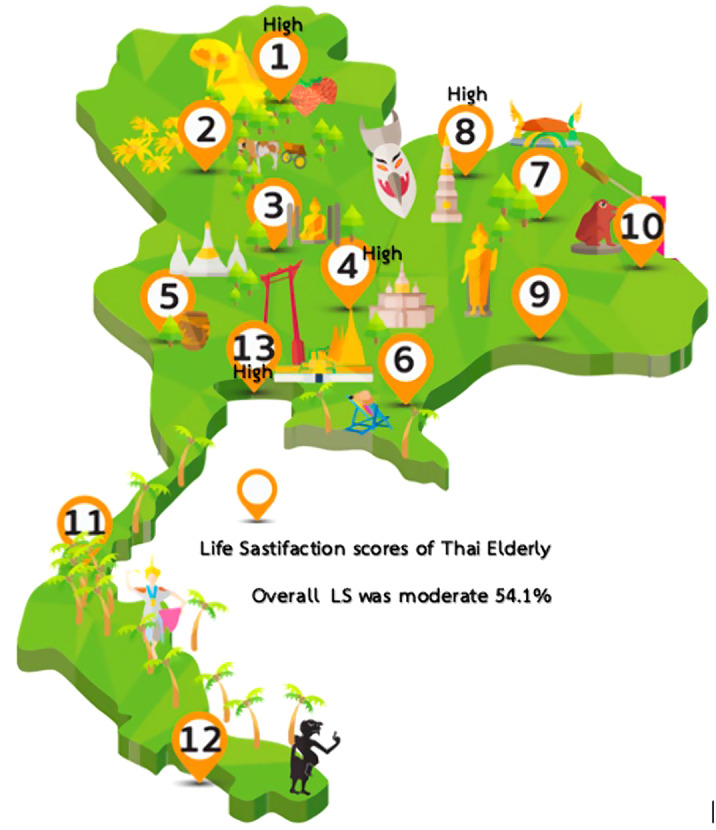

**Figure:**
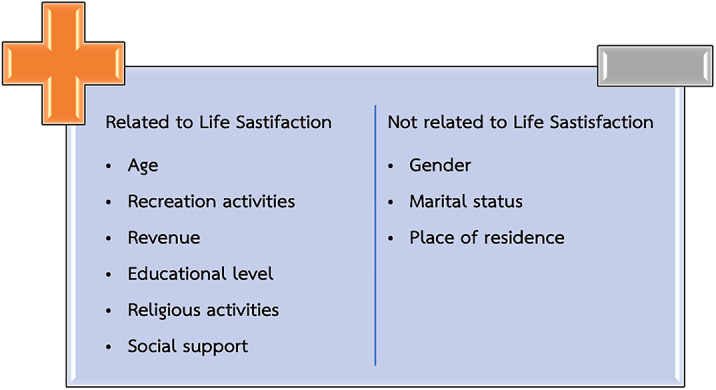

**Figure:**
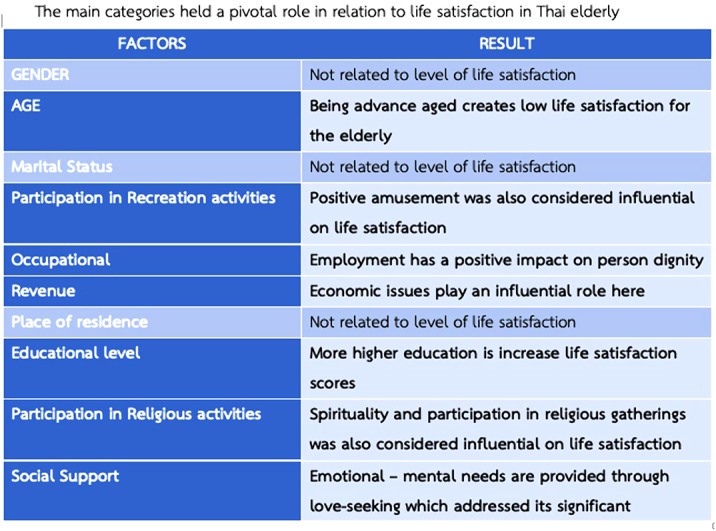

**Conclusions:**

Aging should be foreseen and forethought to increase life satisfaction. The following can be effective in increasing life satisfaction in the elderly: Placing greater emphasis on spiritualism in life, employment of the elderly, and promoting positive leisure in the elderly.

**Disclosure:**

No significant relationships.

